# Q Fever among Culling Workers, the Netherlands, 2009–2010

**DOI:** 10.3201/eid1709.110051

**Published:** 2011-09

**Authors:** Jane Whelan, Barbara Schimmer, Peter Schneeberger, Jamie Meekelenkamp, Arnold IJff, Wim van der Hoek, Mirna Robert–Du Ry van Beest Holle

**Affiliations:** Author affiliations: National Institute for Public Health and the Environment, Bilthoven, the Netherlands (J. Whelan, B. Schimmer, P. Schneeberger, W. van der Hoek, M. Robert–Du Ry van Beest Holle);; European Centre for Disease Prevention and Control, Stockholm, Sweden (J. Whelan);; Jeroen Bosch Hospital, ’s-Hertogenbosch, the Netherlands (P. Schneeberger, J. Meekelenkamp);; ArboUnie, Utrecht, the Netherlands (A. IJff)

**Keywords:** Q fever, epidemiology, agricultural workers’ diseases, occupational diseases, antibodies, bacteria, blood, the Netherlands, culling workers, dispatch

## Abstract

In 2009, dairy goat farms in the Netherlands were implicated in >2,300 cases of Q fever; in response, 51,820 small ruminants were culled. Among 517 culling workers, despite use of personal protective equipment, 17.5% seroconverted for antibodies to *Coxiella burnetii*. Vaccination of culling workers could be considered.

Q fever is caused by the bacterium *Coxiella burnetii.* Since 2007 in the Netherlands, annual outbreaks originating from dairy goat and sheep farms have occurred. In 2009, a total of 2,354 cases in humans were reported, 20% of patients were hospitalized, and at least 6 died (*1*). Among acute cases, ≈2% become chronic, and fatality rates for untreated chronic patients are high (*2*). To stop spread, culling was conducted from December 19, 2009, through June 22, 2010, on 87 infected commercial dairy goat farms and 2 dairy sheep farms (Figure 1). A total of 50,355 pregnant goats and sheep and 1,465 bucks were culled (*3*). Animal pregnancies were confirmed by abdominal ultrasound; pregnant animals were sedated and euthanized, and their corpses were transported to a destruction facility. Culling workers were provided with personal protective equipment (PPE) and advised to read occupational health and hygiene regulations (*4*). To determine seropositivity of workers before culling, incidence of symptomatic and asymptomatic *C. burnetii* infection during culling, and risk factors associated with occupational exposure, we conducted a prospective cohort study.

**Figure 1:**
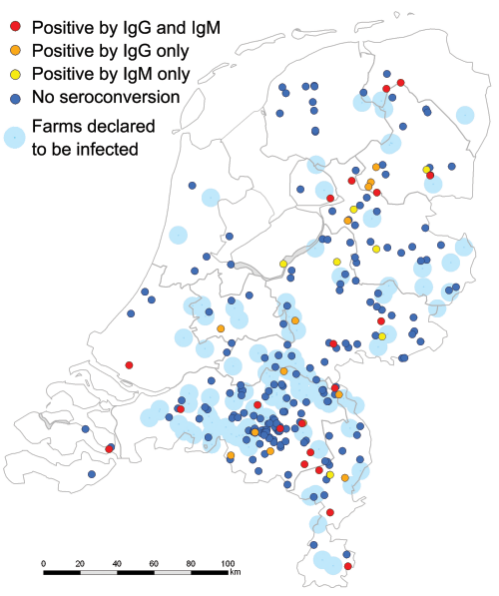
Residential location of 246 culling workers who were seronegative in December 2009 and their serostatus in June 2010 with location of 89 farms declared to be infected (by PCR-positive bulk-milk monitoring) in 2009 and 2010, the Netherlands. Ig, immunoglobulin. Seroconversion detected by ELISA was confirmed by immunofluorescence assay for 40 persons (38 [95%] at titers >128 and 2 [5%] at titers of 32).

## The Study

Participants were 517 workers who culled goats and sheep during December 2009–June 2010. Serum samples were required from workers before employment in December 2009 (pre-cull) (*4*), and voluntary post-cull samples were requested in June 2010. In June, workers were asked to complete a questionnaire about symptoms, occupational exposure, adoption of hygiene measures and PPE use (filtering facepiece masks, gloves, overalls, hairnets), demographics, medical history, and other animal contact. Written informed consent was obtained. Information about farms (animal numbers and abortions) and workers (hours worked per person, job description) was available from occupational records.

Serum was tested for immunoglobulin (Ig) G and IgM against *C. burnetii* phase II by using ELISA (Virion/Serion, Würzburg, Germany). According to manufacturer instructions, IgG phase II seropositivity was defined as negative for titers <30 IU/mL and positive for titers >30 IU/mL. IgM phase II was qualitatively positive or negative. A worker was considered seronegative if a phase II sample was IgM and IgG negative and seropositive if IgM and/or IgG positive. Positive results were confirmed by immunofluorescence assay (Focus Diagnostics, Cypress, CA, USA) titers >32. Symptomatic infection was defined as fever or rigors and >1 of the following after December 1, 2009: malaise, headache, cough, nausea, diarrhea, shortness of breath, pleuritic chest pain, or myalgia. Intensity of occupational exposure was summarized as follows: hours worked, weighted mean farm size (animal number), whether animal abortions were reported, and whether work was performed on average inside or outside the stable (proxy for direct/indirect animal contact). Months worked were dichotomized as cold (December 2009–March 2010) (*5*) and warm (April–June 2010) (*6*). Use of PPE was classified as compliant or noncompliant.

To calculate distance of workers’ residence to the nearest infected farm, we used ArcGIS software (www.esri.com/software/arcgis/index.html). We used

Stata version 11 (StataCorp LP, College Station, TX, USA) to examine univariable associations (Pearson χ^2^ or Fisher exact test). Variables with probability p<0.2 and known risk factors for Q fever were selected for binomial regression analyses. Interactions between significant variables in the multivariable model were investigated. Missing values were excluded.

Of 517 participants, 453 gave pre-cull blood samples, 246 of these gave post-cull samples, and 351 completed the questionnaire. Age, gender, and residential distance from the nearest infected farm were available from occupational records. Participant median age was 47 years (range 19–67 years); 97% were male. Before culling, 14 (3.1%) were IgM II and IgG II positive, 8 (1.8%) were IgM II positive only, 36 (8%) were IgG II positive only, and 395 (87%) were IgG II and IgM II negative; i.e., any seropositivity was found for 13.0%. Pre-cull blood samples indicated more seropositivity among workers who lived within 5 km of an infected farm and had regular work contact with sheep and goats (excluding culling). Prior culling experience was more common among seronegative than seropositive workers (Table 1). Among those who were IgG seropositive before culling, none became IgM seropositive after culling.

**Table 1:** Baseline characteristics of workers before culling small ruminants, the Netherlands, December 2009*

Characteristic	Total no. workers	No. (%) workers		p value†
		Seronegative, n = 395	Seropositive, n = 58	
Sex‡				
M	342	303 (89)	39 (11)	
F§	11	10 (91)	1 (9)	0.812
Age group, y¶				
<40	114	95 (83)	19 (17)	
40–49	157	137 (87)	20 (13)	
50–59	154	139 (90)	15 (10)	
>60	26	22 (85)	4 (15)	0.398
Distance of residence from nearest infected farm, km¶				
<5	116	95 (82)	21 (18)	
>5	317	282 (89)	35 (11)	0.052
Level of education¶				
Low	48	43 (90)	5 (10)	
Medium	132	117 (89)	15 (11)	
High	53	45 (85)	8 (15)	0.725
Medical history¶#				
No	159	140 (88)	19 (12)	
Yes	57	47 (83)	10 (18)	0.288
Current smoker¶				
No	189	162 (86)	27 (14)	
Yes	53	48 (91)	5 (9)	0.357
Previous culling experience¶				
No	116	94 (81)	22 (19)	
Yes	135	124 (92)	11 (8)	0.011
Regular occupational contact with sheep or goats¶				
No	202	182 (90)	20 (10)	
Yes	34	24 (71)	10 (29)	0.002

*Missing values excluded from analysis. †Pearson χ^2^. ‡Maximum 453 respondents. Data available from occupational records. §No female respondents were pregnant. ¶Maximum 251 respondents. Data available from questionnaire responses. #History of cardiorespiratory disease, liver disorders, diabetes, cancer, immunosuppression, allergies, skin conditions.

Among the 395 workers who were seronegative before culling, 246 (62%) provided a follow-up blood sample in June 2010, and 199 (80.8%) of these completed the questionnaire. Those who participated in June were more likely to be male (p = 0.015) and 40–60 years of age (p<0.001). Seroconversion among 246 seronegative respondents occurred as follows: 23 (9.4%) became IgG and IgM seropositive, 7 (2.9%) became IgM positive only, 13 (5.3%) became IgG positive only, and 203 (82.5%) remained seronegative; i.e., any seroconversion was found for 17.5%. Questionnaire respondents who seroconverted had more symptoms after December 1, 2009, (9 [31%] of 29) than nonseroconverters (17 [11%] of 150; relative risk 2.7, 95% confidence interval 1.4–5.5, p = 0.005). Symptomatic seroconverters reported fever and/or rigors and malaise (n = 7), headache (n = 6), cough (n = 6), or myalgia (n = 4). Mean duration of illness was 7.6 (range 1–14) days.

**Figure 2:**
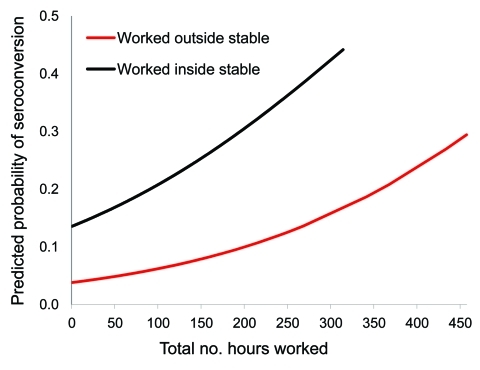
Predicted probabilities of seroconversion among small ruminant culling workers by total hours worked, weighted mean farm size, and location on farm while working during December 2009–June 2010, the Netherlands. Seroconversion probabilities calculated by multivariable model adjusted for age group, occurrence of animal abortions on the farms worked, and compliance with wearing personal protective equipment.

Univariable model indicated significance for total hours worked, farm size, and working inside the stable (p<0.05; Table 2). Multivariable model indicated significance for working >100 hours on the farm and working inside the stable (Table 2; Figure 2). Interaction effects were not significant.

**Table 2:** Variables associated with Q fever seroconversion among 246 workers who were seronegative before culling small ruminants, the Netherlands, 2009*

Variable	No. (%) workers			Univariable analysis			Multivariable analysis†	
	Total	Seroconversion		RR (95% CI)	p value‡		RR (95% CI)	p value
Total	246 (100)	43 (17)						
Sex								
F	6 (2)	2 (33)		Reference				
M	240 (98)	41 (17)		0.51 (0.16–1.64)	0.301			
Age, y								
<45	96 (39)	14 (15)		Reference				
>45	150 (61)	29 (19)		1.33 (0.74–2.38)	0.339		2.0 (0.93–4.16)	0.07
Level of education								
Low	39 (21)	5 (13)		Reference				
Medium	103 (55)	18 (17)		1.36 (0.54–3.42)				
High	44 (24)	8 (18)		1.42 (0.51–3.98)	0.765			
Minimum distance of residence from nearest infected farm, km								
>5	174 (73)	32 (18)		Reference				
<5	63 (27)	9 (14)		0.78 (0.39–1.53)	0.460			
Medical history§								
No	128 (75)	23 (18)		Reference				
Yes	42 (25)	5 (12)		0.66 (0.27–1.63)	0.358			
Current or past smoker								
No	84 (44)	16 (19)		Reference				
Yes	108 (56)	16 (15)		0.78 (0.41–1.46)	0.435			
Total hours worked inside farm perimeter¶								
0–20	81 (33)	5 (6)		Reference			Reference	
21–100	82 (34)	18 (22)		3.56 (1.39–9.12)			5.53 (0.71–42.77)	0.102
>100	80 (33)	20 (25)		4.05 (1.60–10.26)	0.003		7.75 (1.02–58.99)	0.048
Mean farm size >1,500 animals#								
No	167 (68)	22 (13)		Reference				
Yes	79 (32)	21 (27)		2.02 (1.18–3.44)	0.010		1.75 (0.93–3.30)	0.081
Worked mostly inside stable								
No	110 (45)	11 (10)		Reference				
Yes	133 (55)	31 (23)		2.33 (1.23–4.42)	0.006		2.58 (1.04–6.37)	0.040
Animal abortions on farm								
No	208 (85)	33 (16)		Reference				
Yes	38 (15)	10 (26)		1.66 (0.89–3.07)	0.119		0.93 (0.45–1.91)	0.844
Any previous culling experience								
No	85 (43)	15 (18)		Reference				
Yes	114 (57)	17 (15)		0.84 (0.45–1.59)	0.603			
Adherence to hygiene and preventive measures**								
Fully compliant	91 (50)	13 (14)		Reference				
Not compliant	91 (50)	17 (19)		1.31 (0.68–2.53)	0.424		0.94 (0.51–1.72)	0.829
Months spent culling								
Dec 2009–Mar 2010 only (mean temperature 3.2°C)	105 (54)	18 (17)		Reference				
Apr–Jun 2010 only (mean temperature 13.9°C)	2 (1)	1 (50)		2.92 (0.69–12.41)				
Dec 2009–Jun 2010	87 (45)	21 (24)		1.41 (0.80–2.47)	0.288			

*Total for each category may be <246 because of missing data. RR, risk ratio; CI, confidence interval. †All data available for n = 180 in multivariable analysis. ‡Pearson χ^2^. §History of cardiorespiratory disease, liver disorders, diabetes, cancer, immunosuppression, allergies, skin conditions. ¶Data only available for n = 194, those who worked inside the farm perimeter. #Weighted mean number of animals on farms worked by participants. **Includes wearing mask, gloves, overalls, hairnet, showering after exposure.

## Conclusions

Seroconversion for *C. burnetii* among 17.5% of culling workers who were seronegative before culling provides evidence of high-risk work. Before culling, seroprevalence was 13%, similar to that among blood donors in a high-incidence area in the Netherlands in 2009 (H.L. Zaaijer, pers. comm.) and in similar high-risk occupational groups (*7*). Laboratory testing by using ELISA is an accepted method in an acute setting (*8*), and positive results (including positive IgM only) were confirmed by immunofluorescence assay. Nonparticipants were in the youngest and oldest age groups; their effect on the proportion of seroconversion is uncertain. Eighteen workers (excluded for not providing a follow-up blood sample) completed the questionnaire in June. Symptom incidence for these 18 workers was the same as that for included participants.

Symptomatic infection (31% of seroconverters) was probably underestimated. A diagnosis of Q fever was self-reported (unconfirmed) to the occupational health service by 8 workers who did not participate in the study. During December–July 2010, the national infectious disease surveillance system reported 11 culling-related cases of acute Q fever; 2 of these patients were hospitalized.

A strong association was shown between risk for seroconversion and total hours worked on the farms and working inside the stable. In other settings internationally, a risk gradient has also been shown for close direct and indirect animal contact over time (*9**,**10*). In our study, half the participants had experience with previous animal epidemics (avian influenza, foot-and-mouth disease, classical swine fever) and using PPE. Their compliance with PPE was reportedly high; however, a key problem was not wearing PPE while taking work breaks but remaining on the farm.

Given the high risk for infection despite extensive personal protective measures during culling, additional preventive measures are needed. The Health Council of the Netherlands issued guidelines for persons in risk groups who would benefit from vaccination against Q fever (*11*). Culling workers were not included in these guidelines. The efficacy of human Q fever vaccine has been shown to be high for young and healthy persons in similar occupational groups (*12**–**14*). Vaccination of culling workers could be considered if further animal culling is advised.
